# Shear bond strength and debonding characteristics of a new premixed self-etching with a reference total-etch adhesive

**DOI:** 10.1186/s13005-016-0117-x

**Published:** 2016-04-30

**Authors:** Michael Schauseil, Sonja Blöcher, Andreas Hellak, Matthias J. Roggendorf, Steffen Stein, Heike Korbmacher-Steiner

**Affiliations:** Department of Orthodontics, University of Marburg, Georg-Voigt-Str. 3, 35039 Marburg, Germany; Department of Operative Dentistry and Endodontology, University of Marburg, Marburg, Germany

## Abstract

**Background:**

To determine the shear bond strength and adhesive remnant index of a new premixed self-etching primer and adhesive (Tectosan, BonaDent, Germany) for orthodontic appliances in comparison to a reference total-etch system Transbond XT.

**Methods:**

Bovine incisors were embedded in resin and randomly divided into two groups of 16 samples each. Brackets (Discovery, Dentaurum, Germany) were bonded in group 1 (total-etch-system, Transbond XT) and in group 2 (self-etch-system, Tectosan) with curing light for 40 s. Shear bonding strengths were measured after 24 h of storage in distilled water at 37 °C with a Zwicki 1120 testing machine (Zwick Roell, Germany). A force was applied on the bracket base at the wings in occluso-gingival direction. Then the adhesive remnant index (ARI) was determined.

**Results:**

No statistical differences on SBS were found for both bonding agents (p = 0.63). ARI scores however differed statistically significantly (p = 0.035): in the total-etch group more adhesive remained on the teeth, whereas in the self-etch group more adhesive remained on the brackets. There were no visible enamel damages in both groups.

**Conclusions:**

No differences in the shear bond strength were found between both bonding agents. In our study the self-etch-system shifted the adhesive remnant index from more adhesive on the teeth to more adhesive on the bracket - as other already published self-etch systems did - with the new benefit of not increased enamel damages. Tectosan might therefore be a promising alternative to adhesive systems.

## Background

Enhancements of orthodontic materials have improved patient comfort and simplified treatment approaches, especially since time-saving procedures have become an important aspect of effective treatment [1]. Traditional total-etch systems are very technique sensitive and consist of three steps: etching, rinsing and drying. Self-etching primers reduce this process to two (1. Etching + Primer and 2. Bonding) [2] or even to one single-step (Etching + Primer + Bonding) [3]. Because studies in restorative dentistry have shown that self-etch systems can provide comparable results to conventional etching [3], they have also been increasingly used for orthodontic bracket bonding [4, 5]. Although there are studies published saying that self-etching systems are clinically successful [5, 6], there are others stating that they still need improvements to fulfil orthodontic needs of sufficient shear bond strength (SBS) [4]. In the literature usually the required bond strength on adhesive systems ranges between 5.9 and 7.8 MPa [7]: some authors found decreased [8, 9], comparable [10] or even increased [11] SBS values for self-etching primers.

In addition to a sufficient SBS, the breaking point at the adhesive-enamel conjunction during debonding process needs to be evaluated: a greater proximity to the enamel border has the advantage of a fast polishing procedure afterwards but bears the risk of enamel fractures at the same time [10]. Recent studies showed that there was no significant difference in the residual adhesives (Adhesive remnant index = ARI) on the enamel surfaces [12] between different types of bonding. However it needs to be stated that every debonding procedure leads to an individual fracture pattern [13] and in vitro studies showed more bond failures near to the bracket interface [14] what may slow down the polishing process afterwards.

The nature of the adhesive greatly influences the resulting bond strength, the risk of enamel damages and the extent of residual composite on the teeth [14]. Therefore the following demands on an ideal bonding agent can be summarized: short chairtime [15], sufficient SBS (at least within the suggested range) [7], an easy debonding procedure [16] with a breaking line near to the enamel junction [17] and no resulting enamel cracks [18, 19].

A premixed one-bottle-system (Tectosan, BonaDent, Germany) has been recently introduced for bonding of orthodontic appliances [20]. However, there have been no studies published until now comparing the bonding characteristics of it with the standard total-etch system Transbond XT, which is according to the literature the most selected reference for the control group [21, 22].

Other no-mix adhesives decreased the amount of adhesive remnants on the enamel surface but increased enamel damages at the same time [19]. The aim of our in vitro study was therefore to investigate if this new self-etch system has the advantages of the other self-etch systems (high SBS, low ARI score) while not increasing the risk for unwanted enamel damages.

## Methods

For this study we used the method published by Bishara et al. [4] and customized it for our needs. On the basis of ten preliminary measurements (five per group) the needed sample size for the required statistical power was calculated a priori with the software “G*Power for Mac”[23]. Based on the mean values and their standard deviations a needed sample size of 16 per group was calculated. Therefore 32 bovine mandibular incisors were embedded in chemically cured resin. All donor animals were aged between 2 and 5 years. The use of bovine incisors for in vitro tests has already been widely performed in earlier studies and was found to be comparable to human teeth [17, 24, 25]. All teeth were stored in 0.5 % chloramine-T solution for in between 1 week and 6 months, according to the German DIN 1399-1:2009-05. All labial surfaces were positioned upside and parallel to the resin. All teeth were initially polished with Zircate Prophy Paste, rinsed with water and air-dried. Only those teeth were included when enamel surface was free from demineralization, color and structural alterations.

To measure shear bond strength (SBS) the teeth were randomly divided into two groups of 16 samples each (see Table 1):

**Table 1:** Overview of the composition of both test series

	Control group	Experimental group
Etching	37 % phosphoric acid	
Bonding	TransbondXT-primer	Tectosan primer
Adhesive	TransbondXT-adhesive	Tectosan adhesive

- Group 1: Total-etch system Transbond XT, primer and adhesive.
- Group 2: Self-etch system Tectosan, primer and adhesive.

### Bonding procedure

All teeth were bonded with lower premolar brackets (Discovery, Dentaurum, Germany). The average bonding surface of the employed bracket was 13.42 mm^2^. One investigator (XXX) performed the bonding in accordance to the manufacturer’s instructions. All brackets were positioned in the center in between incisal edge and cementoenamel junction.

In the control group the enamel surfaces were etched for 30 s with a 37 % phosphoric acid (Ormco, Orange, CA, USA), rinsed for 10 s with water and air-dried. A thin film of TransbondXT-primer was applied on the etched enamel surface and hardened with a light source for 15 s. TransbondXT-adhesive was applied on the bracket base.

In the experimental group a thin film of self-etching Tectosan-primer was applied on the tooth enamel for ten seconds, followed by drying the surface with air. Tectosan-adhesive was applied on the bracket base.

In both groups the brackets were applied at a pressure of 3 N with the help of a Correx™ gauge (Haag-Streit, Berne, Switzerland), following the procedure described by Bishara et al. [26]. The curing process was conducted in both groups for 20 s with minimal distance each from the mesial and distal side using a light-emitting diode (LED) with a light intensity of 1200 mW/cm^2^ (Elipar™ FreeLight™ 2, 3 M ESPE, Neuss, Germany).

### Debonding procedure

**Fig. 1:**
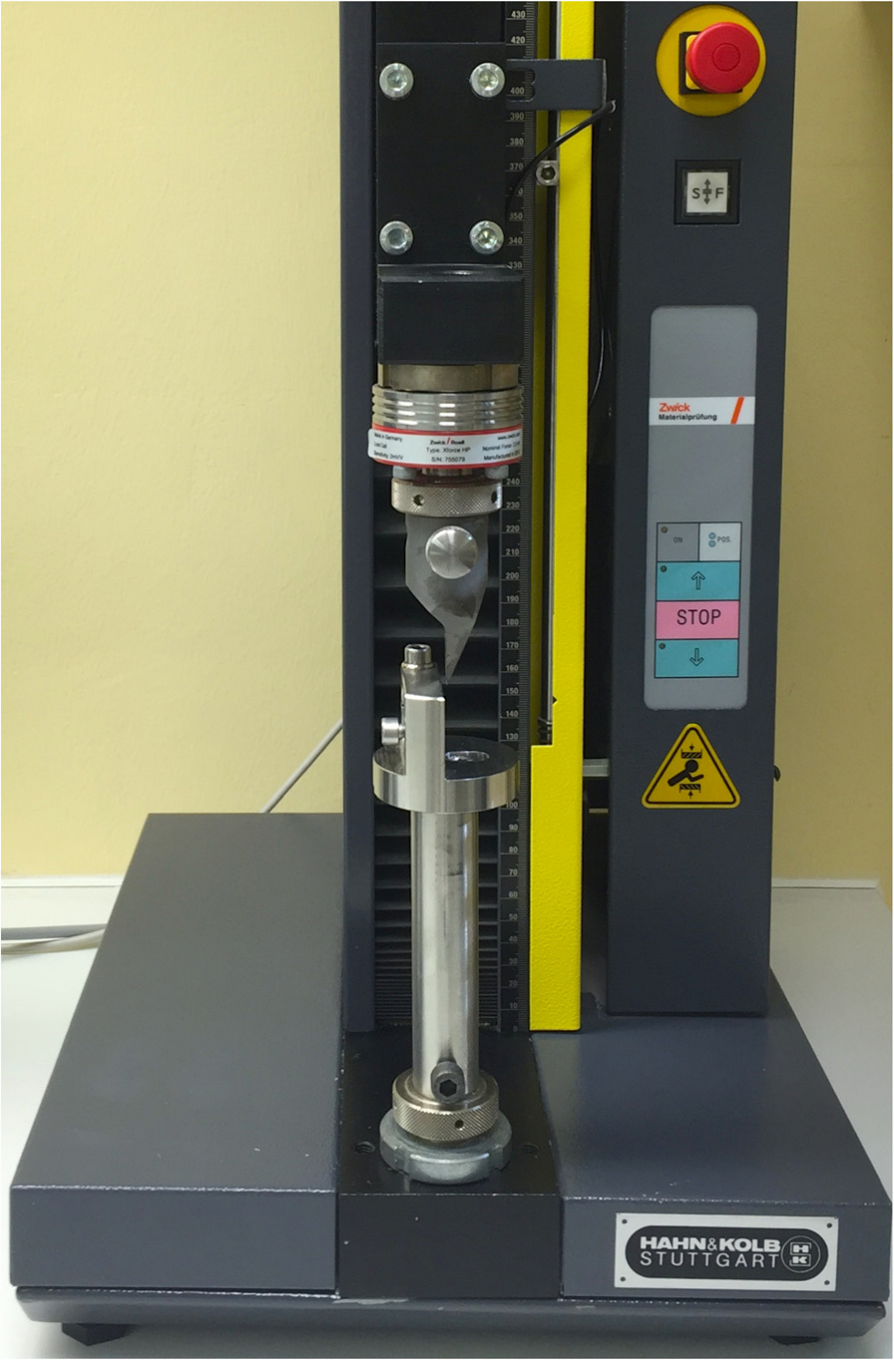
The SBS was measured using a Zwicki 1120 testing machine

Shear bonding strengths were measured after 24 h of storage in distilled water at 37 °C with a Zwicki 1120 testing machine (Zwick Roell, Germany, see Fig. 1). A force was applied on the bracket base at the wings in occluso-gingival direction. The utilized crosshead showed a cuneiform body with a cutting blade on the lower side. Before shear bond procedure the cutting blade was placed parallel, as close as possible without contact to the enamel surface and perpendicular to the upper bracket-base. The bond strength was measured in shear mode at a crosshead speed of 1 mm/min until bracket removal was achieved.

### Residual adhesive

The amount of residual adhesive on the enamel surface was visually measured by using the adhesive remnant index (ARI) score by Årtun and Bergland [27] (for explanation see Table 2).

**Table 2:** The adhesive remnant index (ARI) was measured for every tooth after debonding

ARI-Score	Explanation
0	no adhesive remains on the tooth
1	less than 50 % of the adhesive remains on the tooth
2	more than 50 % of the adhesive remains on the tooth
3	all adhesive remains on the tooth.

All samples were inspected with an optical stereomicroscope (Leica Z 6 APO, magnification 10x, Leica Microsystems, Wetzlar, Germany) [23]. For scanning process the electron microscopy samples were sputtered with gold/platinum in an Edwards sputter coater S150 B (Munich, Germany) and analyzed by SEM image (Phenom FEI G1 and Phenom Software Prosuite, Netherlands). To avoid possible mistakes all ARI scores were determined twice by one investigator after an interval of one week.

### Statistical analysis

Further statistics were calculated using IBM SPSS for Mac, version 21.0 (IBM, New York, USA). Normal distribution was analysed with the help of graphic output and the Shapiro-Wilk test. Because their values were normally distributed SBS was calculated using independent samples *t*-test. Furthermore a Kaplan-Meier survival analysis was performed. Because of no normal distribution the ARI data were analysed using the nonparametric Mann–Whitney test. The significance level for all tests was set at *p* < 0.05.

## Results

### SBS

The mean SBS was 16.59 MPa for reference system and 15.55 MPa for self-etch system, respectively.

The two-tailed *t*-test showed no statistical significant difference (*p* = 0.63) for SBS between both groups (Table 3).

**Table 3:** Shear Bond Strenght of both bonding agents did not differ significantly

Groups	n	mean [MPa]	SD [MPa]	min [MPa]	max [MPa]	*t*-test
Total-etch	16	16.59	6.82	7.03	31.38	*p* = 0.639
Self-etch	16	15.55	5.62	10.15	29.36	

**Fig. 2:**
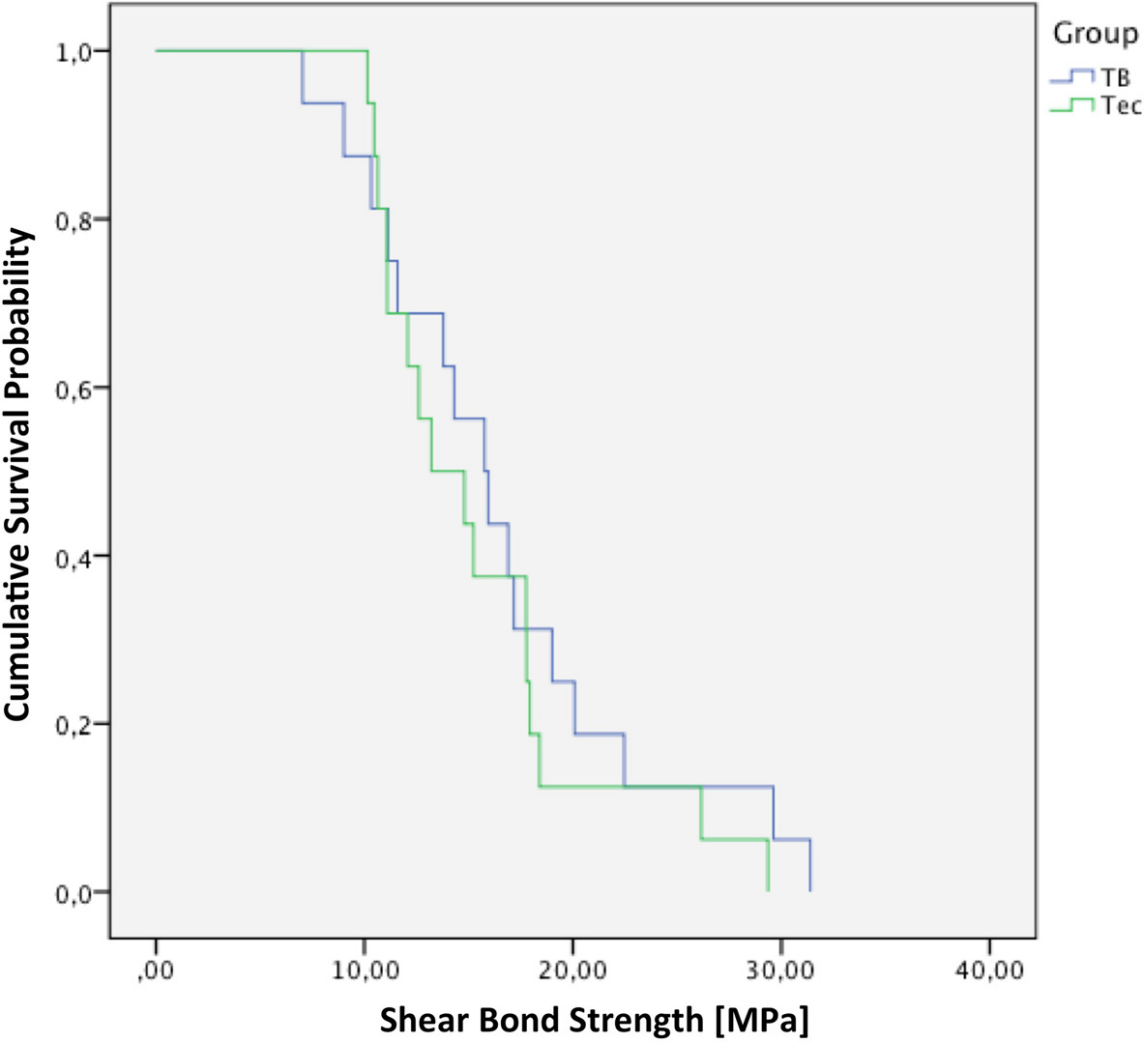
Kaplan-Meier survival analysis for both groups. (TB, total-etch system, Transbond XT; Tec, self-etch system, Tectosan)

Furthermore the Kaplan-Meier graph (Fig. 2) showed that in both groups more than 90 % of the samples showed SBS values above 7.8 MPa.

**Fig. 3:**
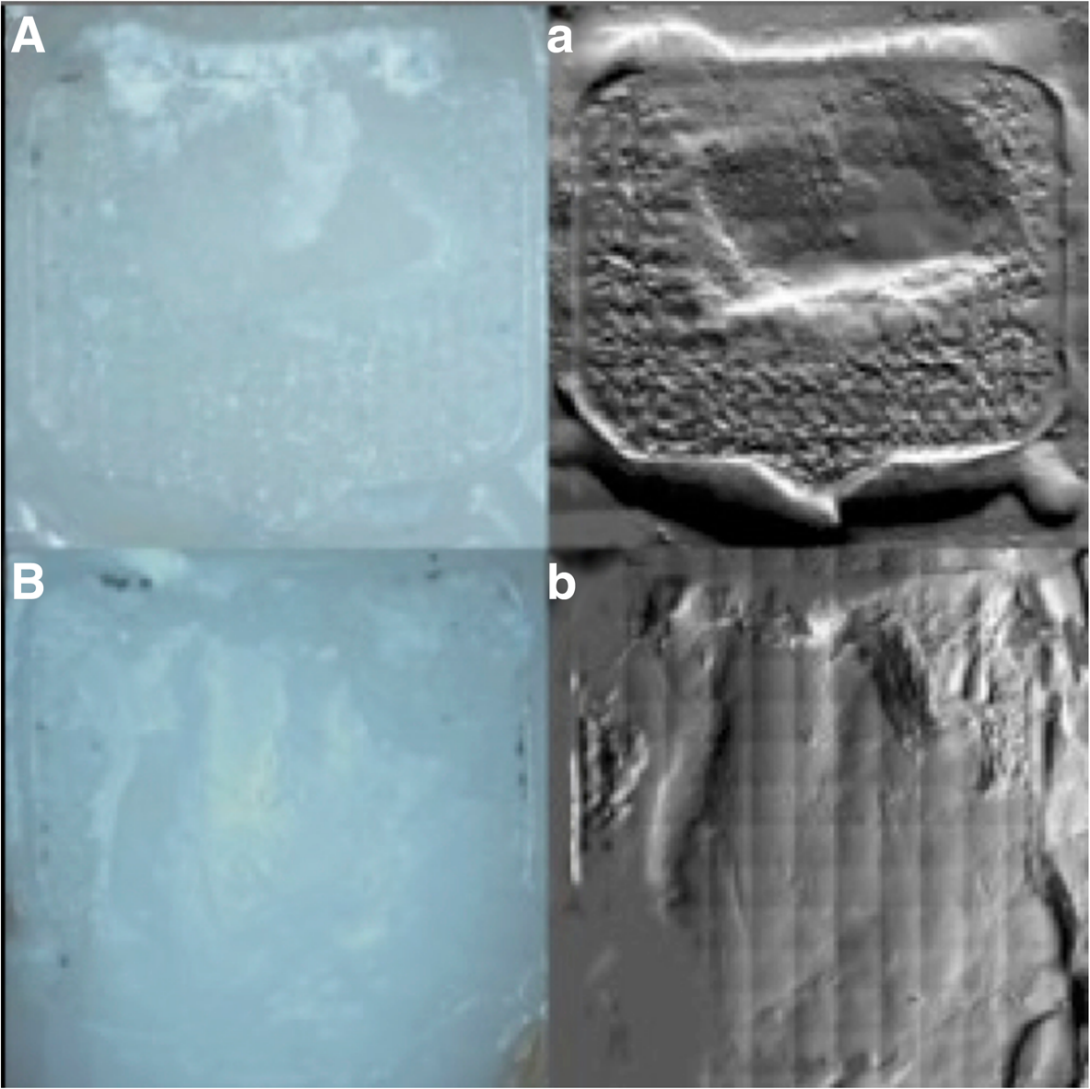
Microscopic and SEM images revealed not any enamel cracks in both groups. **a** and *a*: total-etch system; **b** and *b*: self-etch system. **a** and **b** show a 10x magnification; *a*, and *b* show the SEM counterparts (45x) of the same sample

### ARI

The twofold intraoperator determination of the ARI scores by one investigator showed no differences at all, the applied reproducibility Dahlberg formula got an error of zero [28].

In both groups we found remaining adhesive on all teeth, this is why an ARI score of 0 was not found for any group. However there was not one single visible enamel fracture in both groups.

The two-tailed asymptotic Mann–Whitney test showed a statistical significant difference in the ARI-score between both groups, which was lower after using self-etch system (*p* = 0.035, see Table 4).

**Table 4:** The adhesive remnant index (ARI) on the teeth surfaces for both bonding agents differed significantly

	ARI-Score					Two-tailed Mann–Whitney-*U* test
Groups	0	1	2	3	Median	
Total-etch	0	7	6	3	2	P = 0.035*
Self-etch	0	13	2	1	1	

## Discussion

The risk of debonding-induced enamel defects is related to the bracket system used [17]. Therefore we used only one bracket system which was also used in earlier studies [22] to get comparable results. In our study the self-etch system showed comparable SBS values to those achieved with the reference total-etch system - there was no statistical significant difference for SBS between both groups. The minimal clinically acceptable bond strength ranges between 5.9 and 7.8 MPa [7], which was achieved in both groups.

In this study the ARI scores showed that using the total-etch-system there was significantly more adhesive left on the teeth after debonding (see Fig. 3). This stays in accordance with published in vitro studies comparing total-etch and usual self-etch techniques [9, 29, 30].

The advantage of less adhesive on the teeth is a reduced polishing time, the disadvantage might be an increased risk for enamel fractures [10, 26, 31]. The high SBS and the low ARI, which were found in our study, are consistent with current studies analysing other premixed self-etching primers [19], but in contrast the self-etch system in our study (Tectosan) does not appear to increase the vulnerability to enamel defects: we did not find any cracks on tenfold magnification.

In general, bonding and debonding results in minimal enamel losses, which were found to be higher using a total-etch primer in comparison to a self-etch primer [32, 33]. The minor enamel loss in self-etching systems can be explained with less resting adhesive on the teeth after debonding and thereby less polishing needs afterwards [32]. Because the tested self-etch system showed lower ARI scores a minor enamel loss after debonding might therefore apply for it, too. However, future studies are necessary to prove this fact.

In conclusion it can be stated that this self-etch system seems to provide an acceptable bond strength, which is even greater than the required minimal range, while not leading to higher enamel damages. Further in vivo studies are needed to investigate if these in vitro SBS values will also be found under clinical conditions.

## Conclusions

- Both bonding agends led to a comparable SBS.
- The amount of adhesive on enamel after debonding was significantly less using the self-etch system.
- The tested self-etch system reduces the chair time like other premixed self-etching systems do.
- In contrast to earlier tested self-etching systems this one did not lead to higher enamel damages.
